# Complete genome sequences of *Geobacillus stearothermophilus* strains EF60045 and SJEF4-2 from Korean hot springs

**DOI:** 10.1128/mra.00573-24

**Published:** 2024-08-20

**Authors:** Jae-Yoon Sung, Dariimaa Ganbat, Seong Bo Kim, Sang-Jae Lee, Dong-Woo Lee

**Affiliations:** 1 Department of Biotechnology, Yonsei University, Seoul, South Korea; 2 Department of Bioscience and Research Center for Extremophiles and Marine Microbiology, Silla University, Busan, South Korea; 3 Bio-Living Engineering Major, Global Leaders College, Yonsei University, Seoul, South Korea; SUNY College of Environmental Science and Forestry, Syracuse, New York, USA

**Keywords:** *Geobacillus stearothermophilus*, thermophile, genome, plasmid, hot springs

## Abstract

We report the complete genomes of *Geobacillus stearothermophilus* strains EF60045 and SJEF4-2 from Korean hot springs, with 3,769 and 3,625 thermophilic genes, respectively. *G. stearothermophilus* EF60045 shows four methylation patterns. *G. stearothermophilus* SJEF4-2 harbors three plasmids. These findings enhance understanding of *Geobacillus* strains, aiding in their development as microbial platform hosts.

## ANNOUNCEMENT

*Geobacillus* spp., sourced from Korean hot springs (1), are recognized for their potent enzyme activity and thermostability, making them ideal for industrial applications (2–5). We sequenced the genomes of *Geobacillus stearothermophilus* strains EF60045 and SJEF4-2, isolated from Neungam (37°05'36.5"N 127°48'06.6"E) and Deokgu (37°04'43.8"N 129°16'59.9"E) hot springs. Samples were cultured on marine agar plates (Difco 2216) at 60°C for 5 days to assess their genomic capabilities and biotechnological potential (6).

The *Geobacillus* strains were aerobically cultured in modified Luria-Bertani (LB) medium at 60°C overnight (7). Genomic DNA was extracted using the Wizard Genomic DNA Purification Kit (Promega). For EF60045, DNA was sheared using a g-TUBE (Covaris Inc.) and purified for fragments under 17 kb using AMPurePB magnetic beads (Beckman Coulter Inc.). Sequencing libraries were prepared using the PacBio DNA Template Prep Kit 1.0. SMRTbell (Pacific Biosciences) and sequenced on the PacBio Sequel II platform with Sequel Sequencing Kit 3.0, yielding 78,931 raw reads (651,140,287 bases), which produced a circular consensus sequence averaging 8,249 bp (N_50_ length, 10,526 bp) and compiled a 3,650,785 bp chromosome with 178.0× coverage (Table 1). Further quality enhancement for EF60045 involved aligning short reads generated by the TruSeq Nano DNA Sample Prep Kit (Illumina) using the same genomic DNA. Short-read paired-end libraries (2 × 150 bp) were sequenced on an NovaSeq 6000 instrument (Illumina), yielding 14,759,206 reads. Adapter trimming and quality control were performed using Trimmomatic v0.39 (8) and FastQC v0.11.9 (9), with variant calling via bcftools (“--minDP 5—minQ 30”) (10). Finally, the bcftools consensus function was applied against the pre-assembled contig of *Geobacillus*.

**TABLE 1:** Genomic features of *G. stearothermophilus* strains EF60045 and SJEF4-2

Feature	EF60045	SJEF4-2
Genome size (bp)	3,650,785	3,471,328
No. of contigs	1	1
GC content (%)	52.0	52.5
Total number of genes	3,769	3,625
Protein coding genes (CDS)	3,459	3,310
rRNA genes (5S, 16S, and 23S)	29 (9, 10, and 10)	29 (9, 10, and 10)
tRNA genes	89	88
ncRNA	5	5
Pseudogenes	190	220
CRISPR arrays	5	5
GenBank Accession	CP1298453	CP128449

^*a*^ ncRNA, Non-coding RNA.

SJEF4-2 was sequenced using the Oxford Nanopore MinION platform with the Ligation Sequencing kit SQK-LSK112.24 (Oxford Nanopore Technologies) without DNA shearing or size selection. The sequencing library loaded into the FLO-MIN112 flow cell (R10.4, Oxford Nanopore Technologies) yielded 309,178 reads totaling 1,999,383,409 bp, with a read length N_50_ of 14,234 bp. Adapter trimming and quality control were conducted using Trimmomatic v0.3.2 (8), Nanostat v1.4.0 (11), and FastQC v0.12.0 (9). Flye v2.8.2 (12) with the iteration parameter set to five assembled four circular contigs, with error correction achieved using 25,729,549 Illumina reads for a 519× coverage of the 3,471,328 bp chromosome (Table 1). Additionally, three plasmids—pSJEF4-2-1 (55,393 bp with 727× coverage), pSJEF4-2-2 (13,067 bp with 472× coverage), and pSJEF4-2-3 (5,886 bp with 266× coverage)—were identified. The *dnaA* gene was relocated to the 0 position of the circular genome using the NCBI Prokaryotic Genome Annotation Pipeline (PGAP) to standardize the starting point. The coding sequences of both strains were predicted by the PGAP v6.5 with default parameters (13).

Both strains exhibited larger genomes than the 2.8 Mb genome of ATCC 12980^T^ (GCA_030369615.1), suggesting enhanced carbohydrate-degrading capabilities potentially acquired through horizontal gene transfer (14). Key genes like mtl, abf, and xyn, important for carbohydrate degradation (15, 16), alongside metabolic pathway genes such as pgi, gntK, and acsA, highlight their adaptability and industrial potential (17). This analysis provides a solid foundation for future genetic engineering and utilization of these strains as thermophilic platform hosts (17).

## Data Availability

The whole-genome sequences of *G. stearothermophilus* strains EF60045 and SJEF4-2 have been deposited in the NCBI database under GenBank (BioProject: PRJNA983507) accession numbers CP128453 and CP128449, respectively. Raw sequencing data are available under SRA accession numbers SRR29080532 and SRR29120474 for BioSample SAMN35768001 and SRR29081530 and SRR29081531 for SAMN35766928. Three plasmids from SJEF4-2 are listed under CP128450, CP128451, and CP128452. Methylation motifs for EF60045 are available under SUPPF_0000005608.
